# Haloperidol versus second-generation antipsychotics on the cognitive performance of individuals with schizophrenia and related disorders: pairwise meta-analysis of randomized controlled trials

**DOI:** 10.47626/2237-6089-2023-0664

**Published:** 2025-04-07

**Authors:** Daniel Prates Baldez, Tais Boeira Biazus, Francisco Diego Rabelo-da-Ponte, Guilherme Pedro Nogaro, Dayane Santos Martins, João Pedro Soledade Signori, Vanessa Gnielka, Ives Cavalcante Passos, Letícia Sanguinetti Czepielewski, Maurício Kunz

**Affiliations:** 1 Hospital de Clínicas de Porto Alegre Laboratório de Psiquiatria Molecular Porto Alegre RS Brazil Laboratório de Psiquiatria Molecular, Hospital de Clínicas de Porto Alegre, Porto Alegre, RS, Brazil.; 2 Universidade Federal do Rio Grande do Sul Porto Alegre RS Brazil Programa de Pós-Graduação em Psiquiatria e Ciências do Comportamento, Universidade Federal do Rio Grande do Sul (UFRGS), Porto Alegre, RS, Brazil.; 3 King's College London Institute of Psychiatry, Psychology & Neuroscience Social, Genetic and Developmental Psychiatry Centre London United Kingdom Social, Genetic and Developmental Psychiatry Centre, Institute of Psychiatry, Psychology & Neuroscience, King's College London, London, United Kingdom.; 4 UFRGS Instituto de Psicologia Porto Alegre RS Brazil Programa de Pós-Graduação em Psicologia, Instituto de Psicologia, UFRGS, Porto Alegre, RS, Brazil.

**Keywords:** Cognition, schizophrenia, haloperidol, antipsychotics, meta-analysis

## Abstract

**Objective:**

Despite previous literature, the superiority of second-generation antipsychotics (SGAs) relative to first-generation antipsychotics – especially haloperidol – on cognitive management in schizophrenia is still controversial. Thus, we aimed to compare the effects of haloperidol versus SGAs on the cognitive performance of individuals with schizophrenia or related disorders.

**Methods:**

We conducted an updated systematic review and nine pairwise meta-analyses of double-blinded randomized controlled trials published up to October 30th, 2022, using MEDLINE, Web of Science, and Embase.

**Results:**

Twenty-eight trials were included, enrolling 1,932 individuals. Compared to SGAs, haloperidol performed worse on cognitive composite (mean difference [MD] −0.13; 95% confidence interval [95%CI] −0.33 to −0.03), processing speed (MD −0.17; 95%CI −0.28 to −0.07), attention (MD −0.14; 95%CI −0.26 to −0.02), motor performance (MD −0.17; 95%CI −0.31 to −0.03), memory and verbal learning (MD −0.21; 95%CI −0.35 to −0.08), and executive function (MD −0.27; 95%CI −0.43 to −0.11). In contrast, there were no significant differences between SGAs and haloperidol on working memory (MD 0.10; 95%CI −0.08 to 0.27), visual learning (MD 0.08; 95%CI −0.05 to 0.21), social cognition (MD 0.29; 95%CI −0.30 to 0.88), and visuoconstruction (MD 0.17; 95%CI −0.04 to 0.39).

**Conclusion:**

Haloperidol had poorer performance in global cognition and in some cognitive domains, but with small effect sizes. Therefore, it was not possible to conclude that haloperidol is certainly worse than SGAs in the long-term cognitive management of schizophrenia.

## Introduction

Impairments in cognitive functions are considered a central feature and an important predictor of functionality in schizophrenia.^1,2^ Individuals with schizophrenia are likely to perform poorer in several cognitive domains, including global cognitive scores.^3-5^ The main challenge is establishing pharmacological treatments that effectively improve or reduce cognitive deficits in psychotic disorders. In the last decades, numerous studies have shown that second-generation antipsychotics (SGAs) enhance cognitive performance in patients with psychosis, with better results when compared to first-generation antipsychotics (FGAs).^6-11^ Previous meta-analyses have confirmed the superiority of SGAs, but with a modest-to-moderate effect size.^12-14^

Despite several evidence suggesting SGAs as a better option for long-term treatment in schizophrenia, especially considering their relative superiority to cognitive symptoms, the inferiority of FGAs is still controversial. A meta-analysis published by Mishara and Goldberg^15^ showed that the continued use of FGAs provided significant gains in multiple cognitive domains. Moreover, more extensive clinical trials have also questioned the cognitive superiority of SGAs. The European First Episode Schizophrenia Trial study (EUFEST) analyzed 498 patients with schizophreniform disorder or first-episode schizophrenia and identified a moderate cognitive improvement in the cognitive tests for both SGAs and FGAs, finding no difference in the magnitude of improvement between haloperidol and SGAs.^16^ The Clinical Antipsychotic Trials of Intervention Effectiveness (CATIE), a double-blind randomized controlled trial (RCT) with neuropsychological testing of 817 individuals with schizophrenia, showed a similar effect of perphenazine, a FGAs, compared to olanzapine, risperidone, quetiapine, and ziprasidone.^17^ Therefore, it is unclear the inferiority or non-inferiority of FGAs in cognitive management on psychosis.

Comparing the cognitive effects between FGAs and SGAs is of paramount importance, since both classes were widely used, but with different prevalence around the world. Several low- and middle-income countries keep using FGAs as one of the first options as maintenance treatment in psychotic disorders. The latest World Mental Health Report showed that some SGAs, such as risperidone and clozapine, were only included in less than 35% of national essential medicines lists in low-income countries.^18^ In Brazil, for instance, haloperidol is the main antipsychotic considered essential medicines for public pharmaceutical assistance in the Brazilian Unified Health System (SUS), a national system that ensures access to medicines and health services for the entire population, especially for people with less financial resources.^19^ In contrast, SGAs (clozapine, olanzapine, quetiapine, risperidone, and ziprasidone) are considered specialized medications for pharmaceutical assistance, with more restricted access in the Brazilian public health system.^19^

We previously conducted a systematic review and network meta-analyses to compare the individual effect of 14 antipsychotics on the cognitive performance of individuals with schizophrenia and psychotic disorders.^20^ In this study, we showed that haloperidol has the poorest outcomes in the treatment of cognitive symptoms, but with small effect sizes when compared to SGAs. Thus, considering these unfavorable – and inconclusive – findings, and the widespread use of haloperidol, we designed an updated, complementary analysis to directly compare the cognitive effects of haloperidol and other antipsychotics in the treatment of schizophrenia. The current study extends our previous analyses by assessing whether haloperidol remains with poorer cognitive outcomes even when compared to all other SGAs pooled together. This strategy aims to assess whether haloperidol should be considered a second-line treatment for the cognitive symptoms of schizophrenia.

## Methods

As mentioned above, the present study is a secondary and update analysis of the systematic review and network meta-analyses previously published by our team.^20^ The present study was already described in the original protocol (PROSPERO, number CRD42019142330).

### Systematic review

#### Search strategies

We conducted the systematic review using three databases: MEDLINE (PubMed), Web of Science, and Embase. We first included all studies published up to November 30th, 2018, and we updated data on October 30th, 2022. The search included the following general terms: schizophrenia, psychosis, mood disorder, bipolar disorder, antipsychotic, cognition, memory, attention, working memory, executive function, neuropsychology, and randomized controlled trial. These terms were expanded by the synonym search, and the specific antipsychotic names were also included. We also analyzed all the bibliographic references of the selected studies and all systematic reviews previously published. We followed the Cochrane Handbook for Systematic Reviews of Interventions^21^ and the Preferred Reporting Items for Systematic Reviews and Meta-Analyses (PRISMA) guidelines for systematic reviews and meta-analyses.^22^ We point out that the original search strategy included different psychotic diagnoses to enable comparative analyses between schizophrenia and other disorders. However, in the current analysis, we only included studies related to schizophrenia, excluding studies with patients with bipolar disorder or psychotic depression. The complete search strategies are available in Supplementary Material S1.

#### Inclusion criteria

We included only double-blind RCTs. All studies analyzed individuals between the ages of 18 and 65 diagnosed with schizophrenia or related disorders (schizoaffective disorder and schizophreniform disorder) according to Diagnostic and Statistical Manual of Mental Disorders 3rd edition (DSM-III), 4th edition (DSM-IV), or 4th edition Text Revision (DSM-IV-TR) criteria. We included trials with a follow-up greater than or equal to 4 weeks that compared haloperidol with one or more other antipsychotics – all administered orally. We included studies that measured cognitive performance using neuropsychological tests that considered at least one of the following criteria: (1) the test is completely described in the main compendium of neuropsychology,^23,24^ (2) the test is validated in the main cognitive assessment batteries in schizophrenia,^25-28^ and (3) the test presents a detailed description of its procedures in an article published in a high impact journal.

#### Exclusion criteria

We excluded unblinded trials, co-intervention or adjunct therapy studies, studies with cognitive assessments performed by questionnaires or psychometric scales, trials with participants with neuropsychiatric comorbidities (attention-deficit/hyperactivity disorder, intellectual developmental disorder, and dementia), trials that included individuals with substance-use disorder, and studies that solely examined injectable antipsychotics. We also excluded studies that compared only SGAs versus SGAs, only FGAs versus FGAs, and trials that compared a unique antipsychotic with placebo.

#### Studies’ selection

The screening phase (title and abstracts reading) and eligibility phase (full article reading) were executed independently by two authors (DPB and TBB), and the inconsistencies were analyzed by a third author (FDRP). Data extraction was also carried out by two independent researchers (DPB and GPN). The selections of the cognitive tests were conducted by three trained neuropsychologists (FDRP, DSM, and LSC). The cognitive tests were allocated on cognitive domains by two investigators (FDRP and DSM), also independently, according to the major neuropsychology compendiums,^23,24^ the main cognitive assessment batteries in schizophrenia,^25-28^ and the test definition present in its validation articles (Supplementary Material S2). A third investigator (LSC) analyzed the divergences. We completed the original systematic review in November 2018, but the final analyses were conducted in October 2022 after the update.

### Meta-analyses

Pairwise meta-analyses were carried out to compare the effect of haloperidol and all other antipsychotic agents on cognition. Antipsychotics were primarily classified into FGAs and SGAs,^29^ but we have included drugs from both types. We considered the following cognitive domains: attention, executive function, memory and verbal learning, motor performance, processing speed, social cognition, visual learning, visuoconstruction, and working memory. A cognitive composite score was estimated as described below. The selection of the cognitive domains was based on scientific literature.^23-28^

We performed one meta-analysis for each cognitive domain through the results of cognitive tests (means and standard deviations [SD]) applied in the selected studies. We contacted the study's author in the absence of any published data, and we performed imputation data when the dispersion measures were not available (e.g., SD). The imputation data considered the dispersion measures presented in other included studies (Supplementary Material S3). Studies that evaluated the same sample were grouped as a "single study" to avoid duplication in the statistical analysis. Besides, when different neuropsychological tests referring to a single cognitive domain were applied to the same sample, we considered only the cognitive test with the largest sample size. More details are also presented in the Supplementary Material S3.

In meta-analyses with continuous outcomes, there are different ways of choosing which variable (measure) of a study (trial) will be used for the meta-analysis. We considered the difference (subtraction) between the mean obtained at the study's endpoint and the mean obtained at the study's baseline (Δ or change from baseline) as the measure to be meta-analyzed. We estimated one Δ for each cognitive test applied in each study's arm. The Δ was converted into z-scores (standardized Δ) to allow the results of different tests (with different metrics and units of measure) to be later combined into a single result from a cognitive domain. The SD of Δ was estimated with a correlation index of 0.5.^30^

After measuring the standardized Δ, we calculated the cognitive domain score through the weighted arithmetic average of the standardized Δs, weighted for the number of patients (n) submitted to each test. This weighting was used because we consider that respective tests equally evaluate the cognitive domain. The association between neuropsychological tests and cognitive domains is described in Supplementary Material S2.

We estimated a composite cognitive score for studies that have not previously calculated this measure. The composite score was estimated through the simple arithmetic average of the domains included in the study, giving the same weight to all domains. The composite score was only estimated in studies that evaluated at least the following domains: attention, executive function, memory and verbal learning, processing speed, and working memory. More details are presented in the Supplementary Material S4.

Our meta-analyses were performed in the software R (version 4.2.1), using the package "meta." We used the inverse variance method and the random effect model to calculate the effect sizes, with a 95% confidence interval (95%CI). The summary measures were estimated by mean difference (MD). We did not use the standardized mean difference (SMD) because the results of cognitive tests were previously standardized in z-scores (standardized Δ). The homogeneity was assessed by the Q and I² tests and the similarity was analyzed based on clinical characteristics of the included studies (Supplementary Table S1, available as an Excel file for download). We did not estimate publication bias because none of the direct comparisons included ten or more trials.^31^ The results were presented in forest plots.

The risk of bias and the quality of evidence were assessed by the Cochrane risk of bias 1.0 tool^30^ (Supplementary Material S5). That tool was applied by two independent authors and the disagreements were solved through discussion. The analysis was completed in October 2022.

## Results

**Figure 1:**
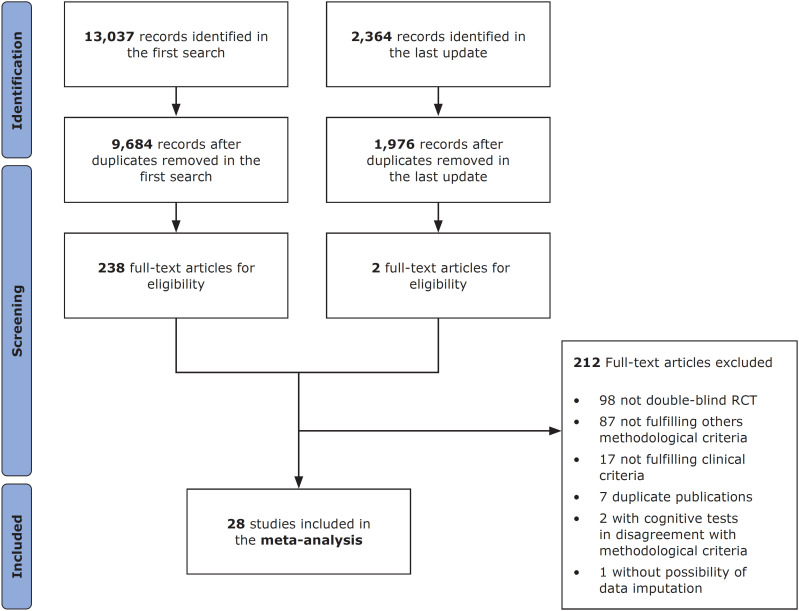
Study selection process. RCT = randomized controlled trial.

The study selection process is shown in Figure 1, the list of included studies is presented in Table 1, and the complete extraction table is presented in Supplementary Table S1 (available as Excel file for download). Briefly, we included 13,037 records in the first search and selected 28 studies for the meta-analysis, comprising 21 independent randomized double-blind controlled trials with 1,932 individuals. In update review, we extracted 2,364 more records; from these, only two articles were included to full-text reading, but none was selected for analysis – we did not find RCTs published from 2020 onwards that met our inclusion criteria. As to the selected studies, 67.29% were multicentered, 64.29% presented a follow-up under 6 months, 92.30% received industry sponsorship, and 89.29% allowed the sporadic use of anticholinergic during the study. Besides, 41.66% included inpatients exclusively, 29.17% included outpatients exclusively, and 29.17% included in and outpatients. We only found RCTs comparing haloperidol versus SGAs. There were no direct comparisons between haloperidol and FGAs.

**Table 1:** Simplified extraction table

First author	M1	M2	M3	M4	M5	M6	M7	M8	M9	M10	Drugs			Follow-up
Abdolahian^32^											Risperidone (n = 35)	Haloperidol (n = 30)		24w
Bilder^6^											Clozapine (n = 24)	Haloperidol (n = 25)	Olanzapine (n = 26)	14w
											Risperidone (n = 26)			
Boulay^33^											Olanzapine (n = 14)	Haloperidol (n = 11)		8w
Buchanan^34^											Clozapine (n = 19)	Haloperidol (n = 19)		10w
Gallhofer^35^											Sertindole (n = 17)	Haloperidol (n = 17)		12w
Green^36^											Risperidone (n = 32)	Haloperidol (n = 30)		2y
Harvey^37^											Risperidone (n = 169)	Haloperidol (n = 169)		12w
Keefe^11^*											Olanzapine (n = 89)	Haloperidol (n = 78)		12w
Keefe^38^*											Olanzapine (n = 18)	Haloperidol (n = 8)		104w
Keefe^10^											Olanzapine (n = 159)	Haloperidol (n = 97)	Risperidone (n = 158)	52w
Green^36^†											Risperidone (n = 30)	Haloperidol (n = 29)		8w
Kee^39†^											Risperidone (n = 9)	Haloperidol (n = 9)		8w
Kern^40†^											Risperidone (n = 27)	Haloperidol (n = 29)		8w
Kern^41†^											Risperidone (n = 32)	Haloperidol (n = 32)		8w
Mcgurk^42†^											Risperidone (n = 28)	Haloperidol (n = 28)		4w
Mcgurk^43†^											Risperidone (n = 26)	Haloperidol (n = 27)		4w
Krakowski^44^											Clozapine (n = 33)	Haloperidol (n = 33)	Olanzapine (n = 34)	12w
Lee^45^											Risperidone (n = 10)	Haloperidol (n = 10)		8w
Lindenmayer^46^											Olanzapine (n = 16)	Haloperidol (n = 19)		12w
Liu^47^											Risperidone (n = 19)	Haloperidol (n = 19)		12w
Purdon^48^											Olanzapine (n = 21)	Haloperidol (n = 23)	Risperidone (n = 21)	54w
Purdon^49^											Quetiapine (n = 13)	Haloperidol (n = 12)		24w
Rémillard^50‡^											Risperidone (n = 15)	Haloperidol (n = 16)		12m
Rémillard^51‡^											Risperidone (n = 14)	Haloperidol (n = 14)		12m
Rosenheck^52^											Olanzapine (n = 159)	Haloperidol (n = 150)		12m
Sergi^53^											Risperidone (n = 40)	Haloperidol (n = 20)	Olanzapine (n = 40)	8w
Smith^54^											Olanzapine (n = 16)	Haloperidol (n = 13)		8w
Velligan^55^											Quetiapine300 (n = 17)	Haloperidol (n=15)	Quetiapine600 (n = 26)	24w

This table shows the complete list of included studies. The 10 meta-analyses are represented in columns M1 to M10, according to the legend below. The studies included in a meta-analysis are highlighted in the respective column. The studies that analyze the same sample are paired with the same symbol (*^†‡^). M1 = processing speed domain; M10 = cognitive composite score; M2 = attention domain; M3 = motor performance domain; M4 = visuoconstruction domain; M5 = memory and verbal learning domain; M6 = visual learning domain; M7 = working memory domain; M8 = executive function domain; M9 = social cognition domain; n = number of individuals included in the study's arm; w = weeks; y = years.

As to the complete sample, 81.19% had a diagnosis of schizophrenia (11.83% had schizoaffective disorder and 6.98% had schizophreniform disorder), 82.14% had previous psychotic episodes, 85.71% had previous history of antipsychotic use, and 67.86% were considered non-refractory to treatment. Moreover, the mean duration of illness was 12.92 years (SD 6.97 years) and the mean age at onset of illness was 24.08 years (SD 7.39 years). Regarding the symptoms’ severity, the sample had an average score of 81.78 (SD 13.98) on Positive and Negative Syndrome Scale (PANSS). We point out that the means described above were estimated considering only the studies that present the respective data. Studies without available data were excluded from the calculation of the percentages and means.

**Figures 2:**
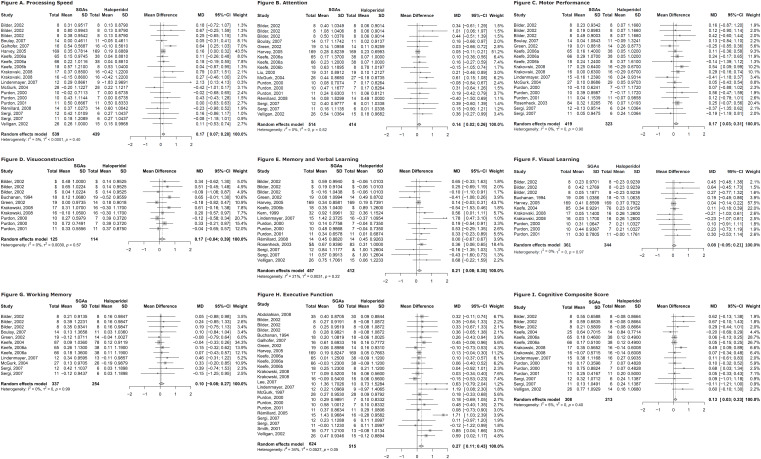
Forest plots for cognitive domains and cognitive composite score. Each figure is a forest plot comparing atypical antipsychotics versus haloperidol in a respective cognitive domain, namely: processing speed (A), attention (B), motor performance (C), visuoconstruction (D), memory and verbal learning (E), visual learning (F), working memory (G), executive function (H), and cognitive composite score (I). In forest plots, each row represents an included clinical trial (for trials with only two arms). When the trial has three or more arms (studies that tested three or more drugs separately), each row represents a possible comparison between the study's drugs, making the same trial occupy more than one row. For trials with three or more arms, all possible pairwise comparisons (between the drugs of each arm) must be performed in the meta-analysis. However, in our analysis, we considered only comparisons that included haloperidol and atypical agent. For instance, if a clinical trial included haloperidol, olanzapine, and quetiapine, we only considered haloperidol versus olanzapine and haloperidol versus quetiapine comparisons, excluding olanzapine versus quetiapine. For trials with three or more arms, the sample size (n) of each drug in a pairwise comparison is estimated by the ratio (division) between the total number of individuals who used the drug and the number of comparisons involving the respective drug. Hypothetically, in a trial with four arms (A, B, C, D), six comparisons are performed (A-B, A-C, A-D, B-C, B-D, C-D). If 90 subjects received drug A, the sample size (n) of drug A in each comparison (A-B, A-C, and A-D) is 30 (90/30). 95%CI = 95% confidence interval; MD = difference between means (or mean difference); mean = continuous variable considered in the metanalysis estimate; SD = standard deviation; SGA = second-generation antipsychotic; total = number of participants; weight = study’ weight in the metanalysis.

The main findings are presented below. The forest plots are presented in Figure 2.

### Processing speed

Fourteen trials were included, with 978 individuals. The mean age was 37.44 years (SD 8.75 years) and 74% males. The analysis included six antipsychotics: clozapine, haloperidol, olanzapine, quetiapine, risperidone, and sertindole. SGAs performed better than haloperidol (MD 0.17; 95%CI 0.07-0.28). The sample showed low and non-significant heterogeneity (I² = 5%; p = 0.40). The results are shown in Figure 2A.

### Attention

Thirteen trials were included, with 928 individuals. The mean age was 38.26 years (SD 8.86 years) and 72.39% males. The analysis included five antipsychotics: clozapine, haloperidol, olanzapine, quetiapine, and risperidone. SGAs performed better than haloperidol (MD 0.14; 95%CI 0.02-0.26). The sample showed non-significant heterogeneity (I² = 0%; p = 0.82). The results are shown in Figure 2B.

### Motor performance

Twelve trials were included, with 742 individuals. The mean age was 38.31 years (SD 8.66 years) and 80.97% males. The analysis included five antipsychotics: clozapine, haloperidol, olanzapine, quetiapine, and risperidone. SGAs performed better than haloperidol (MD 0.17; 95%CI 0.03-0.31). The sample did not show significant heterogeneity (I² = 0%; p = 0.90). The results are shown in Figure 2C.

### Visuoconstruction

Six trials were included, with 239 individuals. The mean age was 35.91 years (SD 8.68 years) and 80.78% males. The analysis included five antipsychotics: clozapine, haloperidol, olanzapine, quetiapine, and risperidone. There was no statistically significant difference between the SGAs and haloperidol (MD 0.17; 95%CI −0.04 to 0.39). The sample did not show significant heterogeneity (I² = 0%; p = 0.57). The results are shown in Figure 2D.

### Memory and verbal learning

Twelve trials were included, with 869 individuals. The mean age was 38.64 years (SD 8.57 years) and 80.42% males. The analysis included five antipsychotics: clozapine, haloperidol, olanzapine, quetiapine, and risperidone. SGAs performed better than haloperidol (MD 0.21; 95%CI 0.08-0.35). The sample showed low and non-significant heterogeneity (I² = 21%; p = 0.22). The results are shown in Figure 2E.

### Visual learning

Seven trials were included, with 705 individuals. The mean age was 32.38 years (SD 8.03 years) and 75.16% males. The analysis included five antipsychotics: clozapine, haloperidol, olanzapine, quetiapine, and risperidone. There was no statistically significant difference between SGAs and haloperidol (MD 0.08; 95%CI −0.05 to 0.21). The sample did not show significant heterogeneity (I² = 0%; p = 0.97). The results are shown in Figure 2F.

### Working memory

Eight trials were included, with 591 individuals. The mean age was 39.47 years (SD 8.63 years) and 77.69% males. The analysis included four antipsychotics: clozapine, haloperidol, olanzapine, and risperidone. There was no statistically significant difference between SGAs and haloperidol (MD 0.10; 95%CI −0.08 to 0.27). The sample did not show significant heterogeneity (I² = 0%; p = 0.99). The results are shown in Figure 2G.

### Executive functions

Eighteen trials were included, with 1,139 individuals. The mean age was 37.17 years (SD 8.29 years) and 75.91% males. The analysis included six antipsychotics: clozapine, haloperidol, olanzapine, quetiapine, risperidone, and sertindole. SGAs performed better than haloperidol (MD 0.27; 95%CI 0.11-0.43). The sample showed moderate heterogeneity but was not statistically significant (I² = 34%; p = 0.05). The results are shown in Figure 2H.

### Social cognition

We found only two clinical trials that met our inclusion criteria. The complete sample (53 subjects) included only three drugs (haloperidol, olanzapine, and risperidone). Therefore, we decided not to perform the meta-analysis for social cognition.

### Cognitive composite score

Nine trials were included, with 521 individuals. The mean age was 37.01 years (SD 8.56 years) and 75.76% males. The analysis included five antipsychotics: clozapine, haloperidol, olanzapine, quetiapine, and risperidone. SGAs performed better than haloperidol (MD 0.13; 95%CI 0.03-0.23). The sample showed low and non-significant heterogeneity (I² = 5%; p = 0.40). The results are shown in Figure 2I.

## Discussion

This study presents the largest meta-analyses comparing the effect of haloperidol and SGAs on the cognitive performance of individuals with schizophrenia. Our results demonstrated poorer performance of haloperidol on cognitive composite score and in the following domains: processing speed, attention, motor performance, memory and verbal learning, and executive function. However, these comparisons had small effect sizes, and there were no statistically significant differences between haloperidol and SGAs on working memory, visual learning, and visuoconstruction.

Previous meta-analyses of clinical studies demonstrated better results to SGAs on cognitive management of schizophrenia and related disorders. Keefe et al.^14^ revealed that SGAs are superior to FGAs to improve cognitive functions in individuals with schizophrenia, especially on verbal fluency, digit-symbol substitution, motor functions, and executive functions. Woodward et al.^12^ also suggested that SGAs are better at improving overall cognitive function, especially processing speed and visual and verbal learning. Guilera et al.^56^ ratified the SGAs’ superiority on the global cognitive index, processing speed, psychomotricity, and language. Désaméricq et al.^13^ showed poorer performance of haloperidol on global score (compared to quetiapine, olanzapine, and risperidone), memory (compared to ziprasidone and olanzapine), attention and processing speed (compared to quetiapine, ziprasidone, olanzapine, and amisulpride), and executive function (compared to quetiapine and olanzapine). Other previous reviews also corroborate these findings. Grada and Dinan^57^ suggested that SGAs had more efficacy in ameliorating inhibition, sustained attention, and set-shifting, all components of executive function. Meltzer et al.^58^ demonstrated that clozapine – a prototype of SGAs – is especially superior to FGAs in some types of cognition, especially verbal fluency. Lee and Park^59^ associated SGAs with better performance in memory and attention.

In contrast, two major RCTs questioned the advantages of SGAs in cognitive performance in schizophrenia. The EUFEST trial also found no differences among haloperidol (FGA) and amisulpride, olanzapine, quetiapine, and ziprasidone on a composite cognitive score.^16^ Despite its large sample size, the EUFEST study had two limitations to be considered: (1) this was an open-label trial, which may have influenced the outcomes; and (2) the cognitive outcomes were assessed by a short cognitive battery, with only five neuropsychological tests, which may not have been able to estimate a global cognition evaluation adequately. The second study was the CATIE trial, that reported no differences in effectiveness between perphenazine (FGA) and olanzapine, quetiapine, risperidone, and ziprasidone (SGAs) on a cognitive composite score, processing speed, reasoning, working memory, verbal memory, and vigilance.^17^

Although our study had shown some unfavorable results for haloperidol, our meta-analysis did not find a worse performance of haloperidol on working memory, visual learning, and visuoconstruction. Previously, Woodward et al.^12^ and Guilera et al.^56^ also did not identify the superiority of SGAs on working memory, visual learning, and visuospatial processing, while Désaméricq et al.^13^ did not test these respective domains. However, our findings are not theoretically grounded in preclinical studies, which tend to demonstrate poor haloperidol results in working memory tasks.^60-63^ Regarding social cognition, it was not possible to perform a meta-analysis because we found only two double-blind RCTs testing antipsychotics’ effects in this domain. A previous study analyzed 15 articles and did not find any conclusive results on the possibility that antipsychotics could specifically facilitate social recovery.^64^ About visuoconstruction, we did not find previous systematic reviews to comparatively evaluate our results.

Our findings should be interpreted considering our limitations and methodological choices. First, in our study, haloperidol showed unfavorable results with small effect sizes. This raises a question about the clinical relevance of our findings, as small statistically significant differences may not be clinically significant. Second, the present study is not theoretically a post-hoc analysis, as it was described *a priori* as a secondary objective of the systematic review in the original protocol. However, we emphasize that all outcomes from secondary objectives have less methodological robustness.

Thirdly, we did not find enough data to assess the dose-dependent effect of haloperidol on cognition (in comparison with SGAs). In previous studies comparing SGAs versus FGAs, there is a recurrent concern that the superiority of the SGAs is justified by the higher doses of the FGAs commonly used in these trials.^65^ However, a previous meta-analysis has already shown that the negative effects of high-dose haloperidol do not explain the cognitive improvements observed with SGAs.^66^ Unfortunately, our review failed to detect the doses’ influence because most of the included trials (19/28 studies) allowed a wide range of haloperidol doses in their samples. Therefore, these trials could not be classified as low-dose (< 12 mg/day) or high-dose (≥ 12 mg/day), which did not enable subgroup analyses. Furthermore, more than half of these trials (15/28) did not present their average antipsychotic daily dose (mg per day), which also did not allow the conduction of secondary analyses.

Fourth, our meta-analyses included studies with a minimum follow-up of 4 weeks, which may be considered short by some authors, but appropriate for others. The minimum follow-up period required for clinical trials to adequately assess the cognitive effects of antipsychotics in schizophrenia is unclear. While Harvey and Keefe^35^ indicate that a 4-week follow-up is sufficient to demonstrate the cognitive effect of antipsychotics and to exclude the effects of previously used medications, the Measurement and Treatment Research to Improve Cognition in Schizophrenia (MATRICS) group suggested longer follow-ups.^67^ Despite the divergences present in the literature, our study is in accordance with the above assumptions.

Fifth, our meta-analyses included individuals at different stages of schizophrenia, indiscriminately, with no specific analysis for each stage of the disease. Thus, our results did not consider the disease's severity as a moderating factor in the effect of antipsychotics on cognition. We could not avoid this limitation because most selected clinical trials gathered patients indistinctly, combining individuals in early stages of the disease and chronic patients. Sixth, we cannot exclude anticholinergics’ influence in our results because most studies did not describe how these drugs were used. This is a relevant limitation, as anticholinergics are associated with cognitive impairment, and the concomitant use of these drugs is more associated with FGAs.^68^

Seventh, we did not consider injectable drugs, such as depot preparations, in our analysis due to pharmacokinetic and pharmacodynamic differences between oral and injectable routes of administration.^29^ In the future, we plan to perform additional analyses focusing exclusively on injectable medications. Eighth, our results may have been significantly influenced by industry bias, as most studies we analyzed were sponsored by pharmaceutical companies. It is important to emphasize that industry bias can exert a powerful influence on the research process.^69^ Finally, due to the small number of RCT designed to assess cognition as a primary outcome in schizophrenia, the results of our meta-analyses are based on secondary outcomes, which reduces the statistical power of our findings.

Our meta-analyses respected statistical and methodological homogeneity assumptions. All meta-analyses obtained results without statistically significant heterogeneity (Q test with p < 0.05), and our screening was able to select trials with methodological and clinical similarities: we only included double-blind RCT, with subjects with a clear diagnosis of schizophrenia, and with no other neuropsychiatric comorbidities, including substance use disorder.

In conclusion, our meta-analyses showed a tendency for haloperidol to present less expressive benefits in the long-term cognitive management in schizophrenia when compared to SGAs. However, it was not possible to conclude that haloperidol is certainly worse than SGAs, because our findings showed small effect sizes, which may not be clinically relevant. Despite our methodological limitations, our results reiterate previous evidence that suggests a possible superiority of SGAs on processing speed, attention, motor performance, memory and verbal learning, executive function, and composite cognition.
