# Supplementary material for: Haloperidol versus second-generation antipsychotics on the cognitive performance of individuals with schizophrenia and related disorders: pairwise meta-analysis of randomized controlled trials
Source: Trends Psychiatry Psychother. 2025 Apr 7;47:e20230664. doi: 10.47626/2237-6089-2023-0664 (PMC12904266; doi:10.47626/2237-6089-2023-0664)
Supplement: Supplementary file 1 [file 2238-0019-trends-47-e20230664-suppl1.pdf]

http://doi.org/10.47626/2237-6089-2023-0664

Supplementary Material S1

Search Strategies

This paper is a complementary analysis of a systematic review previously published by our team[2]. For a complete understanding, we chose to replicate the entire article selection process as supplementary material.

The search was performed using three databases: MEDLINE (PubMed), Web of Science, and EMBASE. We included all studies published up to the initial search date (November 30th, 2018). Two additional strategies were adopted: backward reference searching (analysis of the bibliographic references of the selected studies) and the evaluation of systematic reviews previously published. An updated search was made on November 30th, 2019.

The search terms were initially defined by the researchers and the largest number of synonyms was included. The synonymous terms were identified using the “Mesh Terms” (MEDLINE) and “Emtree” (EMBASE) tools. The general terms are described in the table below, according to the acronym PICOS.

Table. Generic search terms

PICOS*	Search terms[1]
***P**opulation*	schizophrenia<br>psychosis<br>mood disorder<br>bipolar disorder
***I**ntervention*	antipsychotic[2]
***O**utcome*	cognition<br>neuropsychology<br>memory<br>attention<br>working memory<br>executive function
Study design	randomized controlled trial

*PICOS = population, intervention, comparators, outcomes, study design.
[1] These terms were expanded by the synonym search strategy.
[2] Each antipsychotic was named individually in the search strategy

The strategies used in each database are described below. The general terms and their synonyms were grouped by the Boolean operator “OR” and the different general terms were grouped by the Boolean operator “AND”.

I. Search terms and strategy used in MEDLINE

(antipsychotic[Mesh] OR antipsychotic OR antipsychotics OR neuroleptic OR neuroleptics OR aripiprazole[Mesh] OR aripiprazole OR amisulpride[Mesh] OR amisulpride OR benperidol[Mesh] OR benperidol OR asenapine[Mesh] OR asenapine OR blonanserin[Mesh] OR blonanserin OR brexpiprazole[Mesh] OR brexiprazole OR chlorpromazine[Mesh] OR chlorpromazine OR clozapine[Mesh] OR clozapine OR cariprazine[Mesh] OR cariprazine OR clopenthixol[Mesh] OR clopenthixol OR denzapine[Mesh] OR denzapine OR fluanxol[Mesh] OR fluanxol OR fluphenazine[Mesh] OR fluphenazine OR flupenthixol[Mesh] OR flupenthixol OR haldol OR haloperidol[Mesh] OR haloperidol OR iloperidone[Mesh] OR iloperidone OR levomepromazine[Mesh] OR levomepromazine OR lurasidone[Mesh] OR lurasidone OR olanzapine[Mesh] OR olanzapine OR pimozide[Mesh] OR pimozide OR pimavanserin[Mesh] OR pimavanserin OR paliperidone[Mesh] OR paliperidone OR pericyazine[Mesh] OR pericyazine OR perphenazine[Mesh] OR perphenazine OR pipotiazine[Mesh] OR pipotiazine OR prochlorperazine[Mesh] OR prochlorperazine OR promazine[Mesh] OR promazine OR quetiapine[Mesh] OR quetiapine OR risperidone[Mesh] OR risperidone OR sulpiride[Mesh] OR sulpiride OR sultopride[Mesh] OR sultopride OR leuprolide[Mesh] OR leuprolide OR trifluoperazine[Mesh] OR trifluoperazine OR thiothixene[Mesh] OR thiothexe OR zuclopenthixol[Mesh] OR zuclopenthixol OR ziprasidone[Mesh] OR ziprasidone OR zotepine[Mesh] OR zotepine) AND (cognition[Mesh] OR cognition OR neuropsychology[Mesh] OR neuropsychology OR "executive function"[Mesh] OR “executive function” OR "executive functions" OR "inhibitory control" OR "cognitive flexibility" OR "self control" OR "self monitoring" OR "self regulation" OR attention[Mesh] OR attention OR memory[Mesh] OR memory OR "episodic memory" OR "semantic memory" OR "prosodic memory" OR "working

memory"[Mesh] OR working memory) AND ("randomized controlled trial"[pt] OR "controlled clinical trial"[pt] OR randomized[tiab] OR placebo[tiab] OR "drug therapy"[sh] OR randomly[tiab] OR trial[tiab] OR groups[tiab]) AND (Schizophrenia OR Schizophrenia[Mesh] OR Schizophrenias OR “Schizophrenic Disorders” OR “Disorder, Schizophrenic” OR “Disorders, Schizophrenic” OR “Schizophrenic Disorder” OR “Dementia Praecox” OR “Disorder, Paranoid” OR “Disorders, Paranoid” OR “Paranoid Disorder” OR “Psychoses, Paranoid” OR “Paranoid Psychoses” OR “Paranoia” OR “Paranoid Schizophrenias” OR “Schizophrenias, Paranoid” OR “Paranoid Schizophrenia” OR “Delusional Disorder” OR “Delusional Disorders” OR “Disorder, Delusional” OR “Disorders, Delusional” OR Psychosis[Mesh] OR “Disorder, Psychotic” OR “Disorders, Psychotic” OR “Psychotic Disorder” OR “Psychosis” OR “Psychoses” OR “Schizoaffective Disorder” OR “Disorder, Schizoaffective” OR “Disorders, Schizoaffective” OR “Schizoaffective Disorders” OR “Schizophreniform Disorders” OR “Disorder, Schizophreniform” OR “Disorders, Schizophreniform” OR “Schizophreniform Disorder” OR “Psychosis, Brief Reactive” OR “Brief Reactive Psychoses” OR “Brief Reactive Psychosis” OR “Psychoses, Brief Reactive” OR “Reactive Psychoses, Brief” OR “Reactive Psychosis, Brief” OR “Mood Disorder” OR “Mood Disorders” OR “Mood Disorders”[Mesh] OR “Disorder, Mood” OR “Disorders, Mood” OR “Affective Disorders” OR “Affective Disorders”[Mesh] OR “Affective Disorder” OR “Disorder, Affective” OR “Disorders, Affective” OR “Psychoses, Affective” OR “Affective Psychoses” OR “Psychotic Affective Disorders” OR “Affective Disorder, Psychotic” OR “Disorder, Psychotic Affective” OR “Disorders, Psychotic Affective” OR “Psychotic Affective Disorder” OR “Psychotic Mood Disorders” OR “Mood Disorder, Psychotic” OR “Psychotic Mood Disorder” OR “Mood Disorders, Psychotic” OR “Depression, Reactive, Psychotic” OR “bipolar disorder” OR “bipolar disorder”[Mesh] OR “Disorder, Bipolar” OR “Psychosis, Manic-Depressive” OR “Psychosis, Manic Depressive” OR “Manic-Depressive Psychosis” OR “Manic Depressive Psychosis” OR “Affective Psychosis, Bipolar” OR “Bipolar Affective Psychosis” OR “Psychoses, Bipolar Affective” OR “Psychosis, Bipolar Affective” OR “Psychoses, Manic-Depressive” OR “Manic-Depressive Psychoses” OR “mania” OR “manias” OR “Psychoses, Manic Depressive” OR “Manic State” OR “Manic States” OR “State, Manic” OR “States, Manic” OR “Depression, Bipolar” OR “Bipolar Depression” OR “Manic Disorder” OR “Disorder, Manic” OR “Manic Disorders”)

II. Search terms and strategy used in EMBASE

(antipsychotic OR antipsychotics OR neuroleptic OR neuroleptics OR aripiprazole OR amisulpride OR asenapine OR benperidol OR blonanserin OR brexpiprazole OR chlorpromazine OR clozapine OR cariprazine OR clopenthixol OR denzapine OR fenotiazina OR fluanxol OR flupentixol OR fluphenazine OR flupenthixol OR haldol OR haloperidol OR iloperidone OR levomepromazine OR lurasidone OR mosapramine OR olanzapine OR pimavanserin OR paliperidone OR pericyazine OR perospirone OR perphenazine OR pimozide OR pipotiazine OR prochlorperazine OR promazine OR quetiapine OR remoxipride OR risperidone OR sertindole OR sulpiride OR sultopride OR leuprolide OR trifluoperazine OR thiothixene OR thioridazine OR zuclopenthixol OR ziprasidone OR zotepine) AND ('cognition' OR 'neuropsychology' OR 'executive function' OR 'executive functions' OR 'inhibitory control' OR 'cognitive flexibility' OR 'self control' OR 'self monitoring' OR 'self regulation' OR 'attention' OR 'memory' OR 'working memory' OR 'episodic memory' OR 'semantic memory' OR 'prosodic memory') AND ('randomized controlled trial” OR 'controlled clinical trial' OR randomized OR placebo OR 'drug therapy' OR randomly OR trial OR groups) AND (schizophrenia OR schizophrenias OR 'schizophrenic disorders' OR 'disorder, schizophrenic' OR 'disorders, schizophrenic' OR 'schizophrenic disorder' OR 'dementia praecox' OR 'disorder, paranoid' OR 'disorders, paranoid' OR 'paranoid disorder' OR 'psychoses, paranoid' OR 'paranoid psychoses' OR 'paranoia' OR 'paranoias' OR 'paranoid schizophrenias' OR 'schizophrenias, paranoid' OR 'paranoid schizophrenia' OR 'delusional disorder' OR 'delusional disorders' OR 'disorder, delusional' OR 'disorders, delusional' OR 'disorder, psychotic' OR 'disorders, psychotic' OR 'psychotic disorder' OR 'psychosis' OR 'psychoses' OR 'schizoaffective disorder' OR 'disorder, schizoaffective' OR 'disorders, schizoaffective' OR 'schizoaffective disorders' OR 'schizophreniform disorders' OR 'disorder, schizophreniform' OR 'disorders, schizophreniform' OR 'schizophreniform disorder' OR 'psychosis, brief reactive' OR 'brief reactive psychoses' OR 'brief reactive psychosis' OR 'psychoses, brief reactive' OR 'reactive psychoses, brief' OR 'reactive psychosis, brief' OR 'mood disorder' OR 'mood disorders' OR 'disorder, mood' OR 'disorders, mood' OR 'affective disorders' OR 'affective disorder' OR 'disorder, affective' OR 'disorders, affective' OR 'psychoses, affective' OR 'affective psychoses' OR 'psychotic affective disorders' OR 'affective disorder, psychotic' OR 'disorder, psychotic affective' OR 'disorders, psychotic affective' OR 'psychotic affective disorder' OR 'psychotic mood disorders' OR 'mood disorder, psychotic' OR 'psychotic mood disorder' OR 'mood disorders, psychotic' OR 'depression, reactive, psychotic' OR 'bipolar disorder' OR 'disorder, bipolar' OR 'psychosis, manic-depressive' OR 'psychosis, manic depressive' OR 'manic-depressive psychosis' OR 'manic depressive psychosis' OR 'affective psychosis, bipolar' OR 'bipolar affective psychosis' OR 'psychoses, bipolar affective' OR 'psychosis, bipolar affective' OR 'psychoses, manic-depressive' OR 'manic-depressive psychoses' OR 'mania' OR 'manias' OR 'psychoses, manic depressive' OR 'manic state' OR 'manic states' OR 'state, manic' OR 'states, manic' OR 'depression, bipolar' OR 'bipolar depression' OR 'manic disorder' OR 'disorder, manic' OR 'manic disorders') AND ([article]/lim OR [article in press]/lim OR [editorial]/lim OR [letter]/lim OR [note]/lim)[1]

III. Search terms and strategy used in Web of Science

(antipsychotic OR antipsychotics OR neuroleptic OR neuroleptics OR aripiprazole OR amisulpride OR asenapine OR benperidol OR blonanserin OR brexpiprazole OR chlorpromazine OR clozapine OR cariprazine OR clopenthixol

OR denzapine OR fenotiazina OR fluanxol OR flupentixol OR fluphenazine OR flupenthixol OR haldol OR haloperidol OR iloperidone OR levomepromazine OR lurasidone OR mosapramine OR olanzapine OR pimavanserin OR paliperidone OR pericyazine OR perospirone OR perphenazine OR pimozide OR pipotiazine OR prochlorperazine OR promazine OR quetiapine OR remoxipride OR risperidone OR sertindole OR sulpiride OR sultopride OR leuprolide OR trifluoperazine OR thiothixene OR thioridazine OR zuclopenthixol OR ziprasidone OR zotepine) AND TÓPICO: (cognition OR neuropsychology OR "executive function" OR "executive functions" OR "inhibitory control" OR "cognitive flexibility" OR "self control" OR "self monitoring" OR "self regulation" OR attention OR memory OR "working memory" OR "episodic memory" OR "semantic memory" OR "prosodic memory") AND TÓPICO: (randomized controlled trial OR controlled clinical trial OR randomized OR placebo OR drug therapy OR randomly OR trial) AND TÓPICO: (Schizophrenia OR Schizophrenias OR “Schizophrenic Disorders” OR “Disorder, Schizophrenic” OR “Disorders, Schizophrenic” OR “Schizophrenic Disorder” OR “Dementia Praecox” OR “Disorder, Paranoid” OR “Disorders, Paranoid” OR “Paranoid Disorder” OR “Psychoses, Paranoid” OR “Paranoid Psychoses” OR “Paranoia” OR “Paranoias” OR “Paranoid Schizophrenias” OR “Schizophrenias, Paranoid” OR “Paranoid Schizophrenia” OR “Delusional Disorder” OR “Delusional Disorders” OR “Disorder, Delusional” OR “Disorders, Delusional” OR “Disorder, Psychotic” OR “Disorders, Psychotic” OR “Psychotic Disorder” OR “Psychosis” OR “Psychoses” OR “Schizoaffective Disorder” OR “Disorder, Schizoaffective” OR “Disorders, Schizoaffective” OR “Schizoaffective Disorders” OR “Schizophreniform Disorders” OR “Disorder, Schizophreniform” OR “Disorders, Schizophreniform” OR “Schizophreniform Disorder” OR “Psychosis, Brief Reactive” OR “Brief Reactive Psychoses” OR “Brief Reactive Psychosis” OR “Psychoses, Brief Reactive” OR “Reactive Psychoses, Brief” OR “Reactive Psychosis, Brief” OR “Mood Disorder” OR “Mood Disorders” OR “Disorder, Mood” OR “Disorders, Mood” OR “Affective Disorders” OR “Affective Disorder” OR “Disorder, Affective” OR “Disorders, Affective” OR “Psychoses, Affective” OR “Affective Psychoses” OR “Psychotic Affective Disorders” OR “Affective Disorder, Psychotic” OR “Disorder, Psychotic Affective” OR “Disorders, Psychotic Affective” OR “Psychotic Affective Disorder” OR “Psychotic Mood Disorders” OR “Mood Disorder, Psychotic” OR “Psychotic Mood Disorder” OR “Mood Disorders, Psychotic” OR “Depression, Reactive, Psychotic” OR “bipolar disorder” OR “Disorder, Bipolar” OR “Psychosis, Manic-Depressive” OR “Psychosis, Manic Depressive” OR “Manic-Depressive Psychosis” OR “Manic Depressive Psychosis” OR “Affective Psychosis, Bipolar” OR “Bipolar Affective Psychosis” OR “Psychoses, Bipolar Affective” OR “Psychosis, Bipolar Affective” OR “Psychoses, Manic-Depressive” OR “Manic-Depressive Psychoses” OR “mania” OR “manias” OR “Psychoses, Manic Depressive” OR “Manic State” OR “Manic States” OR “State, Manic” OR “States, Manic” OR “Depression, Bipolar” OR “Bipolar Depression” OR “Manic Disorder” OR “Disorder, Manic” OR “Manic Disorders”)

Supplementary Material S2

Allocation of neuropsychological tests in cognitive domains

This paper is an up-date and complementary analysis of a systematic review and meta-analyses previously published by our team[2]. For a complete understanding, we chose to replicate the allocation of neuropsychological test as supplementary material.

The neuropsychological tests were allocated in the cognitive domains by two independent investigators, according to three aspects:

- The test definition present in main neuropsychology compendiums[3,4],
- the test definition present in the main cognitive evaluation batteries in schizophrenia[5,6,7,8]
- The test definition present in its validation article[9].

Neuropsychological tests were allocated to one or more cognitive domains, depending on the researchers' decision. We considered that all tests equally evaluated a respective domain, except:

1. The animal naming test and the letter fluency test were grouped as a single score (verbal fluency), using the simple arithmetic average. This strategy was performed to verbal fluency did not have a greater weight in the calculation on executive function.

2. The tests that assessed the verbal memory domain were first categorized into three subgroups: short-term verbal memory, long-term verbal memory, and verbal learning. After estimating the result of each subgroup (z-scores), we estimated the simple arithmetic mean of these results to obtain the *memory and verbal learning* score. We carried out this strategy to the three subgroups had the same weight in the final estimate.

http://doi.org/10.47626/2237-6089-2023-0664

Table S2-I. The allocation of neuropsychological tests in cognitive domains

Neuropsychological tests (and measures)	Cognitive domain
Continuous Performance Test – Identical Pairs - d-prime	Attention
Continuous Performance Test - degraded-stimulus	Attention
D2 Cancellation test/ D2 Test of Attention – errors	Attention
Digit span - Forward (recall the digits in the correct order) - percentages	Attention
Digit span distractibility task	Attention
Identification Test - Cogstate[8]	Attention
Rapid Visual Information Processing – total hits, total errors - CANTAB[9]	Attention
Span of Apprehension – error score	Attention
Stroop Color-Word Test - correct responses, hit rate, number of errors, false alarms	Attention
Wechsler Memory Scale – Revised - Visual Memory Span - forward	Attention
Design Fluency Test	Executive function
Groton Maze Learning Test - Cogstate[8]	Executive function
Maze tests	Executive function
Ruff Figural Fluency Test	Executive function
Self-ordered pointing tasks/ Subjective ordered pointing tasks – errors	Executive function
Stroop Test – interference	Executive function
Stockings of Cambridge – problems solved on first choice and mean choices to correct - CANTAB[9]	Executive function
Trail Making Test – Part B – time to completion	Executive function
Tower of London – correct responses and execution time	Executive function
Verbal fluency – Category Fluency (animal naming) – correct responses	Executive function
Verbal fluency – Letter fluency (COWAT) – correct responses	Executive function
Wisconsin Card Sorting Test - perseverative errors, total errors, number of categories completed	Executive function
Continuous Performance Test – Identical Pairs – reaction time	Processing speed
Detection Test – Cogstate[5]	Processing speed
Digit Symbol/ Symbol Coding – correct responses	Processing speed
Reaction time – correct responses - CANTAB[9] and similar tests	Processing speed
Stroop Color-Word Test – reaction time, speed of naming, colors stripes	Processing speed
Trail Making Test – Part A – time to completion	Processing speed
Continuous Paired Associate Learning Tasks – Cogstate[8]	Working memory
Digit sequencing test – correct responses	Working memory
Digit span – Backward – percentages	Working memory
Letter–Number Sequencing Test/ Letter–number span test – number of correct trials	Working memory
One-Back Memory Task and Two-Back Memory Task – Cogstate[8]	Working memory
Peterson Consonantes Trigram Test	Working memory
Spatial reference memory test – delayed 5 seconds and delayed 15 seconds – errors	Working memory
Spatial working memory test – CANTAB[9]	Working memory
Spatial Working Memory Test – 5 sec and 15sec	Working memory
Visuospatial working memory	Working memory
Wechsler Memory Scale – III – Spatial Span Test –backward	Working memory
Wechsler Memory Scale – Revised – Visual memory span – backward	Working memory
Finger Tapping Test – number of taps	Motor performance
Grooved Pegboard – number of pegs successfully inserted	Motor performance
Motor Screening Test – mean errors – CANTAB[9]	Motor performance
Pin Test – total number	Motor performance
Rey-Osterrieth Complex figure test – copy	Motor performance
Rey-Taylor complex figure test- copy	Motor performance
Rey-Taylor complex figure test	Motor performance

Token motor task – number of tokens correctly placed	Motor performance
Logical memories task – number of recalled cues	Long-term verbal memory
Paragraph recall – delayed recall total	Long-term verbal memory
Rey Auditory Verbal Learning Test (RAVLT), Hopkins Verbal Learning Test-Revised (HVLT), California Verbal Learning Test (CVLT) – long-delay recall (CVLT), delayed recall – trial 7/ A7 (RAVLT)	Long-term verbal memory
Verbal recognition memory – delayed recall – correct responses - CANTAB[9]	Long-term verbal memory
Wechsler Memory Scale – Revised – Logical Memory – delayed – correct responses	Long-term verbal memory
Auditory Comprehension Test – story recall	Short-term verbal memory
Rey Auditory Verbal Learning Test (RAVLT), Hopkins Verbal Learning Test-Revised (HVLT), California Verbal Learning Test (CVLT) – trial 1/ list A	Short-term verbal memory
Verbal recognition memory – immediate recall – correct responses - CANTAB[9]	Short-term verbal memory
Wechsler Memory Scale – Revised – Logical Memory – immediate – correct responses	Short-term verbal memory
International Shopping List Task – Cogstate[8]	Verbal learning
Rey Auditory Verbal Learning Test (RAVLT), Hopkins Verbal Learning Test-Revised (HVLT), California Verbal Learning Test (CVLT) – Total number of words recalled correctly over three learning trials (HVLT), over trials 1 – 5 (RAVLT), and learning trial 1-5 (CVLT)	Verbal learning
Rey and Crawford Auditory Verbal Learning Tests	Verbal learning
Serial digital learning/ Digit Sequence Learning/ Benton Serial Learning Test	Verbal learning
Hooper visual Organization Test	Visuoconstruction
Wechsler Adult Intelligence Scale – Revised (WAIS-R) – Block Design – Total number of points, age-corrected	Visuoconstruction
Wechsler Adult Intelligence Scale – III (WAIS-III) – Object assembly	Visuoconstruction
Wechsler Adult Intelligence Scale – III (WAIS-III) Picture Completion	Visuoconstruction
Brief Visuospatial Memory Test-Revised (BVMT) – Total recall score over three learning trials	Visual learning
Design list learning/ Rey Design Learning Test/ Serial Design Learning Test	Visual learning
Paired Associates Learning – total errors - CANTAB[9]	Visual learning
Pattern Recognition Memory – immediate and delayed (correct responses and percentages) - CANTAB[9]	Visual learning
Rey-Osterrieth Complex figure test – total recall and immediate recall	Visual learning
Rey-Taylor complex figure test – immediate recall	Visual learning
Visual Learning Test – CogSate[8]	Visual learning
Wechsler Memory Scale – Revised – Visual pairs – total number of correct word associations	Visual learning
Wechsler Memory Scale – Revised – immediate recall	Visual learning
Wechsler Memory Scale – Revised – delayed recall	Visual learning
Wechsler Memory Scale – Revised – Figural memory – correct responses	Visual learning
Face Emotion Discrimination Test (FEDT) – correct responses	Social cognition
Facial Emotion Identification Test - correct responses	Social cognition
Half-Profile of Nonverbal Sensitivity - correct responses	Social cognition
Interpersonal Perception Task – correct responses	Social cognition
Maryland Assessment of Social Competence	Social cognition
Penn Emotional Acuity Test – correct responses	Social cognition
Social Cue Recognition Test - sensitivity	Social cognition
Social Emotional Cognition Test – Cogstate[8]	Social cognition
Social Skills Performance Assessment - total	Social cognition
Voice Emotion Identification Test – correct responses	Social cognition

Supplementary Material S3

Imputation of missing data

This paper is a complementary analysis of a systematic review previously published by our team[2]. For a complete understanding, we chose to replicate the imputation data as supplementary material.

Some studies included in our meta-analyses did not describe the measures of dispersion (standard deviation, standard error, or confidence interval). In these cases, these measures were imputed from other included studies. The imputation was performed “between results of the same cognitive test, in the same unit of measurement”. When a study presented the results of a cognitive test without the measures of dispersion, these values were imputed from another study that applied the same test (in the same measure).

Considerations:

- The imputation was not performed in the absence of another study that applied the same test and in the same unit of measurement.
- When several studies applied the same cognitive test, we chose the largest measure of dispersion as the measure to be used for imputation. This is the most conservative strategy for imputation data.
- In our study, we performed imputations in six included studies. They are described in the analysis table. The analysis table can be requested from the corresponding author.

Some studies presented their results only graphically. We used the *Web Plot Digitizer*[10] to extract the graphic data (mean and measures of dispersion). The *Web Plot Digitizer* is a website available from: https://apps.automeris.io/wpd/. All the results were checked manually by two independent researchers.

Supplementary Material S4

Calculation and standardization of cognitive test scores, cognitive domain scores, and cognitive composite cognitive

This paper is a complementary analysis of a systematic review previously published by our team[2]. For a complete understanding, we chose to replicate the calculation of cognitive scores as supplementary material.

We considered the result of a cognitive test as the difference between the mean obtained at study’s endpoint and the mean obtained at study’s baseline (Δ or *mean difference* or *change from baseline*).

$$\Delta = \bar{x}_{\text{endpoint}} - \bar{x}_{\text{baseline}}$$

Δ = mean difference (change from baseline)
$\bar{x}_{\text{endpoint}}$ = mean at endpoint
$\bar{x}_{\text{endpoint}}$ = mean at baseline

In the studies that did not present a *mean difference* (Δ), we estimated the standard deviation of Δ according to the following equation, using a correlation index of 0.5.

$$SD\Delta = \sqrt{(sd_{baseline})^2 + \left(sd_{endpoint}\right)^2 - 2\ corr\ x\ sd_{baseline} x\ sd_{endpoint}}$$

SD_{Δ} = standard deviation of Δ
sd $_{\text{baseline}}$= standard deviation at baseline
sd $_{\text{endpoint}}$ = standard deviation at endpoint
corr = correlation index

The result of a cognitive test (*mean difference*) were standardized by their standard deviation at baseline. Thus, we obtained the test results in z-scores.

$$\Delta \text{ standardized } = \frac{\Delta}{\text{sd baseline}}$$

Δ standardized = standardized mean differences
Δ = mean differences
$sd_{baseline}$ = standard deviation at baseline.

Likewise, the standard deviation of Δ was standardized by the standard deviation at baseline.

$$\text{SD } \Delta \text{ standardized } = \frac{\text{SD } \Delta}{\text{sd baseline}}$$

SDΔ standardized = standardized standard deviation
SDΔ = standard deviation of Δ
$sd_{baseline}$ = standard deviation in baseline

The cognitive domain score was estimated through the weighted arithmetic average of the standard scores from its respective cognitive tests, weighted for the sample size (*n*) of each test. This condition did not cause any harm to the analysis for considering that all tests equally evaluate the cognitive domain.

$$\bar{x}_n = \frac{\bar{x}_1 . n_1 + \bar{x}_2 . n_2 + \cdots + \bar{x}_n . n_n}{n_t}$$

$\bar{x}_n$ = domain score
x_1 = standardized average of test 1
n = number of patients tested in test 1
n_t = total number of patients assessed in the domain

The composite score was calculated through the simple arithmetic average of the domains present in the study. The simple arithmetic average allowed that all cognitive domains had the same weigh in the global cognitive estimative.

$$\bar{x}_n = \frac{\bar{x}_1 . + \bar{x}_2 + \bar{x}_3 + \ \dots + \bar{x}_{11}}{n_t}$$

$\bar{x}_n$ = composite cognitive score
x_1 = standardized average of domain 1
n = number of patients tested assessed in the composite score.
n_t = total number of patients

Considerations:

1. We estimated a cognitive composite score only from studies that evaluated, at least, the following domains: executive function, memory, verbal learning, work memory, processing speed, and attention. We judged inappropriate the estimate of a composite cognitive score in the absence of these domains.

2. To estimate the standard deviation of the composite score, we considered the same sample size (*n*) for all domains, with the smallest *n* among the domains.

3. Some studies have presented the cognitive test results in more than one unit of measurement. In such cases, we performed the weighted average between them. For instance, if a study evaluated the Wisconsin Card Sorting Test through the measures of persevering errors and

completed categories, we performed the weighted average of these outcomes. Afterwards, this estimate was included in the calculation of the executive function score.

4. An increase in the scores of a cognitive test could represent an improvement or a worsening. To gather the different tests into a single score (cognitive domain), it was necessary a standardization. Thus, to estimate a final test score, we maintained or changed its signs according to the meaning of this finding. If the result symbolized an improvement, we did not change the sign; if the final score symbolized a worsening, the sign was reversed. For instance, in the measurement of perseverative errors in the Wisconsin Card Sorting Test, an increase of this value represents a worsening. So, if the result found is -0.2 (the number of errors decreased by 0.2 z-scores), the negative sign is transformed to positive (because the reduction of errors designates improvement). The same rule dictated the elaboration of the global cognitive score.

Supplementary Material S5

Cochrane Risk of Bias

We assessed the risk of bias from included clinical trials using the Cochrane Risk of Bias 1.0 tool[11]. The Cochrane Handbook for Systematic Reviews of Interventions can be found at https://training.cochrane.org/handbook/current. For more details, we provide the supplementary table nº 6.

Table S5-I. The Cochrane Collaboration's tool for assessing risk of bias

**Domain**	**Support for judgement**	**Review authors' judgement**
Selection bias.		
**Random sequence generation.**	Describe the method used to generate the allocation sequence in sufficient detail to allow an assessment of whether it should produce comparable groups.	Selection bias (biased allocation to interventions) due to inadequate generation of a randomized sequence.
**Allocation concealment.**	Describe the method used to conceal the allocation sequence in sufficient detail to determine whether intervention allocations could have been foreseen in advance of, or during, enrolment.	Selection bias (biased allocation to interventions) due to inadequate concealment of allocations prior to assignment.
Performance bias.		
**Blinding of participants and personnel** Assessments should be made for each main outcome (or class of outcomes).	Describe all measures used, if any, to blind study participants and personnel from knowledge of which intervention a participant received. Provide any information relating to whether the intended blinding was effective.	Performance bias due to knowledge of the allocated interventions by participants and personnel during the study.
Detection bias.		
**Blinding of outcome assessment** Assessments should be made for each main outcome (or class of outcomes).	Describe all measures used, if any, to blind outcome assessors from knowledge of which intervention a participant received. Provide any information relating to whether the intended blinding was effective.	Detection bias due to knowledge of the allocated interventions by outcome assessors.
**Incomplete outcome data** Assessments should be made for each main outcome (or class of outcomes).	Describe the completeness of outcome data for each main outcome, including attrition and exclusions from the analysis. State whether attrition and exclusions were reported, the numbers in each intervention group (compared with total randomized participants), reasons for attrition/exclusions where	Attrition bias due to amount, nature or handling of incomplete outcome data.

	reported, and any re-inclusions in analyses performed by the review authors.	
Reporting bias.		
**Selective reporting.**	State how the possibility of selective outcome reporting was examined by the review authors, an what was found.	Reporting bias due to selective outcome reporting.
Other bias.		
**Other sources of bias.**	State any important concerns about bias not addressed in the other domains in the tool. If particular questions/entries were pre-specified in the review's protocol, responses should be provided for each question/entry.	Bias due to problems not covered elsewhere in the table.

Table S5-II. Risk of bias table of included studies

	Sequence generation	Allocation concealment	Blinding participants and prescribers	Blinding assessor	Incomplete outcome data	Selective reporting
Abdolahian, 2008	Unclear	Unclear	Unclear	Unclear	Unclear	Unclear
Bilder, 2002	Unclear	Unclear	Low risk	Unclear	Unclear	Unclear
Boulay, 2007	Unclear	Unclear	Unclear	Unclear	Low risk	Unclear
Buchanan, 1994	Unclear	Unclear	Low risk	Low risk	Low risk	Unclear
Galhofer, 2007	Unclear	Unclear	Unclear	Unclear	Low risk	Unclear
Green, 1997	Low risk	Low risk	Unclear	Unclear	Low risk	Unclear
Green, 2002	Unclear	Unclear	Unclear	Unclear	High risk	Unclear
Harvey, 2005	Low risk	Low risk	Unclear	Unclear	Unclear	Unclear
Kee, 1998	Low risk	Low risk	Unclear	Unclear	Unclear	Unclear
Keefe, 2004	Unclear	Unclear	Unclear	Unclear	High risk	High risk
Keefe, 2006a	Unclear	Unclear	Unclear	Unclear	High risk	Unclear
Keefe, 2006b	Unclear	Unclear	Unclear	Unclear	Low risk	Unclear
Kern, 1998	Low risk	Low risk	Unclear	Unclear	Unclear	Unclear
Kern, 1999	Low risk	Low risk	Unclear	Unclear	Unclear	Unclear
Krakowski, 2008	Low risk	Unclear	Low risk	Low risk	Low risk	Unclear
Lee, 2007	Unclear	Unclear	Unclear	Low risk	Unclear	Unclear
Lindenmayer, 2007	Unclear	Unclear	Low risk	Unclear	Unclear	Unclear
Liu, 2000	Low risk	Unclear	Unclear	Unclear	High risk	Unclear
McGurk, 1997	Low risk	Low risk	Unclear	Unclear	Unclear	Unclear
McGurk, 2004	Low risk	Low risk	Unclear	Unclear	Unclear	Unclear
Purdon, 2000	Low risk	Low risk	Low risk	Unclear	Low risk	Unclear
Purdon, 2001	Unclear	Unclear	Unclear	Unclear	Low risk	Unclear
Rémillard, 2005	Unclear	Unclear	Unclear	Low risk	Unclear	Unclear
Rémillard, 2008	Unclear	Unclear	Unclear	Low risk	Unclear	Unclear
Rosenheck, 2003	Low risk	Unclear	Low risk	Unclear	Low risk	High risk
Sergi, 2007	Low risk	Low risk	Unclear	Unclear	High risk	Unclear
Smith, 2001	Unclear	Unclear	Unclear	Unclear	High risk	Unclear
Velligan, 2002	Unclear	Unclear	Unclear	Unclear	High risk	Unclear

http://doi.org/10.47626/2237-6089-2023-0664

Supplementary Material S6

List of included studies in the systematic review and meta-analyses

1. Abdolahian E, Mohareri F, Bordbar MRF: Haloperidol versus risperidone: A comparison of beneficial effect on cognitive function of patients with chronic schizophrenia. Iran J Psychiatry Behav Sci 2008; 2:14–20
2. Bilder RM, Goldman RS, Volavka J, et al.: Neurocognitive effects of clozapine, olanzapine, risperidone, and haloperidol in patients with chronic schizophrenia or schizoaffective disorder. Am J Psychiatry 2002; 159:1018–1028
3. Boulay LJ, Labelle A, Bourget D, et al.: Dissociating medication effects from learning and practice effects in a neurocognitive study of schizophrenia: Olanzapine versus haloperidol. Cogn Neuropsychiatry 2007; 12:322–338
4. Buchanan RW, Holstein C, Breier A: The comparative efficacy and long-term effect of clozapine treatment on neuropsychological test performance. Biol Psychiatry 1994; 36:717–725
5. Gallhofer B, Jaanson P, Mittoux A, et al.: Course of recovery of cognitive impairment in patients with schizophrenia: A randomised double-blind study comparing sertindole and haloperidol. Pharmacopsychiatry 2007; 40:275–286
6. Green MF, Marshall BD, Wirshing WC, et al.: Does risperidone improve verbal working memory in treatment-resistant schizophrenia? Am J Psychiatry 1997; 154:799–804
7. Green MF, Marder SR, Glynn SM, et al.: The neurocognitive effects of low-dose haloperidol: A two-year comparison with risperidone. Biol Psychiatry 2002; 51:972–978
8. Harvey PD, Rabinowitz J, Eerdekens M, et al.: Treatment of cognitive impairment in early psychosis: A comparison of risperidone and haloperidol in a large long-term trial. Am J Psychiatry 2005; 162:1888–1895
9. Kee KS, Kern RS, Marshall BD, et al.: Risperidone versus haloperidol for perception of emotion in treatment-resistant schizophrenia: Preliminary findings. Schizophr Res 1998; 31:159–165
10. Keefe RSE, Seidman LJ, Christensen BK, et al.: Comparative effect of atypical and conventional antipsychotic drugs on neurocognition in first-episode psychosis: a randomized, double-blind trial of olanzapine versus low doses of haloperidol. Am J Psychiatry 2004; 161:985–995
11. Keefe RSE, Young CA, Rock SL, et al.: One-year double-blind study of the neurocognitive efficacy of olanzapine, risperidone, and haloperidol in schizophrenia. Schizophr Res 2006; 81:1–15
12. Keefe RSE, Seidman LJ, Christensen BK, et al.: Long-term neurocognitive effects of olanzapine or low-dose haloperidol in first-episode psychosis. Biol Psychiatry 2006; 59:97–105
13. Kern RS, Green MF, Marshall BD, et al.: Risperidone vs. haloperidol on reaction time, manual dexterity, and motor learning in treatment-resistant schizophrenia patients. Biol Psychiatry 1998; 44:726–732
14. Kern RS, Green MF, Marshall BDJ, et al.: Risperidone versus haloperidol on secondary memory: Can newer medications aid learning? Schizophr Bull 1999; 25:223–232
15. Krakowski MI, Czobor P, Nolan KA: Atypical antipsychotics, neurocognitive deficits, and aggression in schizophrenic patients. J Clin Psychopharmacol 2008; 28:485–493
16. Lee S-M, Chou Y-H, Li M-H, et al.: Effects of antipsychotics on cognitive performance in drug-naive schizophrenic patients. Prog Neuro-Psychopharmacology Biol Psychiatry 2007; 31:1101–1107
17. Lindenmayer J-P, Khan A, Iskander A, et al.: A randomized controlled trial of olanzapine versus haloperidol in the treatment of primary negative symptoms and neurocognitive deficits in schizophrenia. J Clin Psychiatry 2007; 68:368–379
18. Liu SK, Chen WJ, Chang CJ, et al.: Effects of atypical neuroleptics on sustained attention deficits in schizophrenia: A trial of risperidone versus haloperidol. Neuropsychopharmacology 2000; 22:311–319
19. McGurk SR, Green MF, Wirshing WC, et al.: The effects of risperidone vs haloperidol on cognitive functioning in treatment-resistant schizophrenia: the Trail Making Test. CNS Spectr 1997; 2:60–64
20. McGurk SR, Green MF, Wirshing WC, et al.: Antipsychotic and anticholinergic effects on two types of spatial memory in schizophrenia. Schizophr Res 2004; 68:225–233
21. Purdon SE, Jones BDW, Stip E, et al.: Neuropsychological change in early phase schizophrenia during 12 months of treatment with olanzapine, risperidone, or haloperidol. Arch Gen Psychiatry 2000; 57:249–258
22. Purdon SE, Malla A, Labelle A, et al.: Neuropsychological change in patients with schizophrenia after treatment with quetiapine or haloperidol. J Psychiatry Neurosci 2001; 26:137–149
23. Rémillard S, Pourcher E, Cohen H: The effect of neuroleptic treatments on executive function and symptomatology in schizophrenia: A 1-year follow up study. Schizophr Res 2005; 80:99–106
24. Rémillard S, Pourcher E, Cohen H: Long-term effects of risperidone versus haloperidol on verbal memory, attention, and symptomatology in schizophrenia. J Int Neuropsychol Soc 2008; 14:110–118
25. Rosenheck R, Perlick D, Bingham S, et al.: Effectiveness and Cost of Olanzapine and Haloperidol in the Treatment of Schizophrenia. J Am Med Assoc 2003; 290:2693–2702
26. Sergi MJ, Green MF, Widmark C, et al.: Social Cognition and Neurocognition: Effects of Risperidone, Olanzapine, and Haloperidol. Am J Psychiatry 2007; 164:1585–1592
27. Smith RC, Infante M, Singh A, et al.: The effects of olanzapine on neurocognitive functioning in medication-refractory schizophrenia. Int J Neuropsychopharmacol 2001; 4:239–250

28. Velligan DI, Newcomer J, Pultz J, et al.: Does cognitive function improve with quetiapine in comparison to haloperidol? Schizophr Res 2002; 53:239–248

Supplementary Material S7

List of studies excluded in the final phase of the systematic review.

I. Studies excluded due to lack of data and impossibility of imputation

1. Jean Addington P, Donald Addington M: Neurocognitive Functioning in Schizophrenia: A Trial of Risperidone Versus Haloperidol. Can J Psychiatry 1997; 42:983

II. Studies excluded for applying only neuropsychological tests in disagreement with our methodological criteria

1. Strauss WH, Klieser E, Luethcke H: Dyscognitive syndromes in neuroleptic therapy. Pharmacopsychiatry 1988; 21:298–299
2. Saletu B, Kiifferle B, Grünberger J, et al.: Quantitative EEG, SPEM, and Psychometrie Studies in Schizophrenics before and during Differential Neuroleptic Therapy. Pharmacopsychiatry 1986; 19:434–437

References

1 Page MJ, McKenzie JE, Bossuyt PM, Boutron I, Hoffmann TC, Mulrow CD et al. The PRISMA 2020 statement: an updated guideline for reporting systematic reviews. British Medical Journal. 2021:n21.
2 Baldez DP, Biazus TB, Rabelo-da-Ponte FD, Nogaro GP, Martins DS, Kunz M, et al. The effect of antipsychotics on the cognitive performance of individuals with psychotic disorders : Network meta-analyses of randomized controlled trials. Neuroscience and Biobehavioral Reviews. 2021;126:265–75.
3 Strauss E, Sherman EMS, Spreen O. A compendium of neuropsychological tests: Administration, norms, and commentary. (3rd ed.). Oxford University Press. 2006.
4 Lezak MD, Howieson DB, Bigler ED, Tranel D. Neuropsychological assessment (5th ed.). Oxford University Press. 2012.
5 Keefe RSE, Goldberg TE, Harvey PD, Gold JM, Poe MP, Coughenour L. The Brief Assessment of Cognition in Schizophrenia: Reliability, sensitivity, and comparison with a standard neurocognitive battery. Schizophr Res. 2004;68:283–97.
6 Keefe RSE, Mohs RC, Bilder RM, Harvey PD, Green MF, Meltzer HY, et al. Neurocognitive assessment in the clinical antipsychotic trials of intervention effectiveness (CATIE) project schizophrenia trial: Development, methodology, and rationale. Schizophr Bull. 2003;29:45–55.
7 Nuechterlein KH, Green MF, Kern RS, Baade LE, Barch DM, Cohen JD, et al. The MATRICS consensus cognitive battery, part 1: Test selection, reliability, and validity. Am J Psychiatry. 2008;165:203–13.
8 Pietrzak RH, Olver J, Norman T, Piskulic D, Maruff P, Snyder PJ. A comparison of the CogState Schizophrenia Battery and the Measurement and Treatment Research to Improve Cognition in Schizophrenia (MATRICS) Battery in assessing cognitive impairment in chronic schizophrenia. J Clin Exp Neuropsychol. 2009;31:848–59.
9 Ernst Nielsen R, Odur F, Østergaard T, Munk-Jørgensen P, Nielsen J. Comparison of the effects of Sertindole and Olanzapine on Cognition (SEROLA): A double-blind randomized 12-week study of patients diagnosed with schizophrenia. Therapeutic Advances in Psychopharmacology. 2014;4:4–14.
10 Rohatgi A. WebPlotDigitizer. 2020. Available at https://automeris.io/WebPlotDigitize.
11 Higgins JPT, Altman DG, Gøtzsche PC, Jüni P, Moher D, Oxman AD, et al. The Cochrane Collaboration's tool for assessing risk of bias in randomised trials. Br Med J. 2011.
